# The Tobacco Industry’s Role in the 16 Cities Study of Secondhand Tobacco Smoke: Do the Data Support the Stated Conclusions?

**DOI:** 10.1289/ehp.9385

**Published:** 2006-08-29

**Authors:** Richard L. Barnes, S. Katharine Hammond, Stanton A. Glantz

**Affiliations:** 1 Center for Tobacco Control Research and Education, University of California, San Francisco, California, USA; 2 Cardiovascular Research Institute and Department of Medicine, University of California, San Francisco, California, USA; 3 School of Public Health, University of California, Berkeley, California, USA

**Keywords:** 16 Cities Study, Center for Indoor Air Research, environmental tobacco smoke, ETS, Occupational Safety and Health Administration, OSHA, R.J. Reynolds Tobacco Co., Roger Jenkins, secondhand tobacco smoke, SHS, smoke-free homes, smoke-free workplaces

## Abstract

**Background:**

Since 1996, the tobacco industry has used the 16 Cities Study conclusions that workplace secondhand tobacco smoke (SHS) exposures are lower than home exposures to argue that workplace and other smoking restrictions are unnecessary.

**Objectives:**

Our goal was to determine the origins and objectives of the 16 Cities Study through analysis of internal tobacco industry documents and regulatory agency and court records, and to evaluate the validity of the study’s conclusions.

**Results:**

The tobacco industry’s purpose in conducting the 16 Cities Study was to develop data showing that workplace SHS exposures were negligible, using these data to stop smoking restrictions by the U.S. Occupational Safety and Health Administration. The extensive involvement of R.J. Reynolds Tobacco Company and the tobacco industry’s Center for Indoor Air Research in controlling the study was not fully disclosed. The study’s definition of “smoking workplace” included workplaces where smoking was restricted to designated areas or where no smoking was observed. This definition substantially reduced the study’s reported average SHS concentrations in “smoking workplaces” because SHS levels in unrestricted smoking workplaces are much greater than in workplaces with designated smoking areas or where no smoking occurred. Stratifying the data by home smoking status and comparing exposures by workplace smoking status, however, indicates that smoke-free workplaces would halve the total SHS exposure of those living with smokers and virtually eliminate SHS exposure for most others.

**Conclusions:**

Data in the 16 Cities Study reveal that smoke-free workplaces would dramatically reduce total SHS exposure, providing significant worker and public health benefits.

The tobacco industry has a history of manipulating science regarding both active and passive smoking to serve its political, legal, and regulatory needs (Barnes and Bero 1998; Barnoya and Glantz 2002, 2005; Bero 2005b; Bero et al. 1994; Glantz et al. 1996; Hirschhorn and Bialous 2001; Hong and Bero 2002; Muggli et al. 2001; Ong and Glantz 2001). Public concern about the health effects of secondhand smoke (SHS) increased sharply in 1981 when Hirayama (1981) linked SHS to lung cancer in non-smokers, followed by the 1986 Surgeon General’s Report, *The Health Consequences of Involuntary Smoking* [U.S. Department of Health and Human Services (DHHS) 1986] and National Research Council (NRC) reports on SHS (NRC 1986b) and airliner cabin air quality (NRC 1986a). Just as it did when confronted with evidence in the 1960s that smoking caused lung cancer in smokers (Bero et al. 1995; Glantz et al. 1996), the tobacco industry responded with research designed to “establish a controversy” about the evidence linking SHS with disease (Barnes and Bero 1996, 1997, 1998; Barnes et al. 1995; Barnoya and Glantz 2002, 2005; Bero 2003, 2005b; Bero et al. 1994, 2001, 2005; Hirschhorn and Bialous 2001; Hong and Bero 2002; Muggli et al. 2001; Ong and Glantz 2001; Schotland and Bero 2002). This effort included industry personnel designing and supervising studies that undermined the connection between SHS and lung cancer (Lee 1995) and contested the conclusion that there were substantial levels of toxicants in airliners where smoking was allowed (Malmfors et al. 1989). Both these reports were published under the names of nonindustry authors and minimized unfavorable (to the tobacco industry) results (Hong and Bero 2002; Neilsen and Glantz 2003; Yano 2005).

In 1988, in response to a citizen petition seeking a standard prohibiting smoking in indoor workplaces (Public Citizen 1987), the U.S. Occupational Safety and Health Administration (OSHA) obtained a written analysis of the literature on SHS (Meridian Research 1988) that reported that the literature, though compelling, relied on data from residential exposures only and concluded, “What seems unequivocally clear at this time, is the need to resolve these issues by means of personal sampling to ascertain the relative contribution of workplace exposure to a non-smoker’s overall exposure to environmental tobacco smoke from all sources.” OSHA did not act on this recommendation or make the report public.

In June 1991, the National Institute for Occupational Safety and Health (NIOSH) issued the Current Intelligence Bulletin *Environmental Tobacco Smoke in the Workplace: Lung Cancer and Other Health Effects* (NIOSH 1991), which urged employers to protect employees from SHS by prohibiting smoking in all workplaces. This publication was followed in 1992 by the U.S. Environmental Protection Agency (EPA) risk assessment that identified SHS as a Group A (human) carcinogen (U.S. EPA 1992). These reports, combined with continued pressure from health advocates (Bryan-Jones and Bero 2003; Public Citizen 1987), led OSHA in 1994 to propose a workplace standard on indoor air quality that included substantial restrictions on indoor smoking (OSHA 1994). OSHA assumed that SHS exposure levels at work would be at least as high as those in homes. The actual levels of SHS exposure were a potentially important issue in the OSHA rule-making because, at the time, risk estimates for lung cancer were based on studies of non-smoking spouses married to smokers.

The tobacco industry responded with the “16 Cities Study” (Jenkins et al. 1996), which was specifically conceived and designed to oppose the OSHA regulation. It was the first large study using personal monitoring equipment to sample airborne SHS constituents in both workplaces and worker homes and to compare those exposures to each other. The study was designed by R.J. Reynolds Tobacco Company (RJR) scientists, and all of the field work and laboratory analyses for the study were done by RJR personnel. RJR exercised substantial control of the research throughout its conduct and analysis. The 16 Cities Study presentations to OSHA (Jenkins 1994a, 1994b, 1994c, 1995a; Jenkins and Guerin 1994) and published 1996 paper (Jenkins et al. 1996) did not disclose the full involvement of RJR and other tobacco industry scientists. The study concluded that home SHS exposures were two to four times greater than workplace exposures. The purpose of the 16 Cities Study was to affect policy making, not to advance scientific knowledge; the results as presented (Jenkins et al. 1996) served the tobacco industry’s goal of preventing regulation of smoking. A careful review of the 16 Cities data, however, reveals that smoking was restricted in most of the “smoking workplaces” and that few cigarettes were observed being smoked there. Presenting the data in a way that accounts for actual smoking in workplaces reveals, in contrast to the interpretation presented by the tobacco industry and the researchers it funded, that requiring smoke-free workplaces would cut the total SHS exposure of those living with smokers in half and all but eliminate SHS exposure for those living in nonsmoking homes, providing a significant worker safety and public health benefit.

## Methods

Between September 2004 and October 2005 we searched tobacco industry documents and Deposition and Trial Testimony Archive (DATTA) transcripts in the University of California, San Francisco Legacy Tobacco Documents Library (http://legacy.library.ucsf.edu and http://ltdlftd.library.ucsf.edu/), the British American Tobacco Document Archive (http://bat.library.ucsf.edu), and Tobacco Documents Online (http://tobac-codocuments.org). Initial search terms were Roger Jenkins, 16 Cities, and reference (Bates) numbers near relevant documents. After identifying the first documents, we used a snowball strategy to locate new documents. A total of about 500 relevant documents were reviewed.

We used the Access World News Internet newspaper archives (http://infoweb.newsbank.com) and other Internet resources to find information on administrative hearings and actions and on tobacco industry lawsuits in which Roger Jenkins was involved as a witness or potential witness, and the transcripts of the 1995 OSHA hearings on the proposed Indoor Air Quality Standard (OSHA 1995).

We used tabulated data from the 16 Cities Study (Jenkins and Counts 1999; Jenkins et al. 1996) to illustrate that a different analysis (stratification by smoking policy) and presentation of the results would lead to different conclusions from those the tobacco industry and its consultants reported.

## Results

Table 1 lists key events related to the origin, conduct, and use of the 16 Cities Study.

### The origin of the 16 Cities Study

At its 11 April 1991 meeting, the Tobacco Institute (TI) Executive Committee (TI 1991c) approved an “EPA/OSHA Strategic Plan” (TI 1991b) for opposing U.S. EPA and OSHA action on secondhand tobacco smoke. (TI was the tobacco industry’s political and lobbying organization.) The plan sought to prevent release of the U.S. EPA risk assessment of SHS (U.S. EPA 1992) and to forestall SHS legislation at federal, state, and local levels. At the August 1991 TI Executive Committee meeting, separate U.S. EPA and OSHA project scientific research recommendations (TI 1991a) were presented (TI 1991d) by Susan Stuntz, TI Vice President for Public Relations, and John Rupp, a Covington & Burling attorney who served as counsel to the Institute and played a key role in a wide range of SHS issues (Barnoya and Glantz 2002; Drope and Chapman 2001; Muggli et al. 2001, 2003, 2004). The stated objective of the “OSHA Projects” scientific research was to “encourage adoption of a ventilation standard and to discourage adoption of a smoking ban or of a standard that requires separate ventilation for areas where smoking is allowed” (TI 1991a). This objective fits into the industry’s overall strategy of presenting ventilation as an alternative to smoke-free environments (Bialous and Glantz 2002; Bryan-Jones and Bero 2003; Drope et al. 2004). Among the four research projects the report recommended to support this objective was one to “develop data demonstrating ... that ETS [environmental tobacco smoke, the term the tobacco industry developed for SHS] exposures in the typical workplace are too low to support the notion of a significant risk” and “to compile for the first time all available data on levels of ETS and other pollutants in the home, in the workplace and in social settings” (TI 1991a).

The only large SHS exposure study (Turner et al. 1992) available at that time (funded secretly by the tobacco industry) was conducted by Healthy Buildings International (HBI) and used area sampling in 585 offices (Barnes et al. 1995; Turner et al. 1992). HBI was a frequent contributor to the industry’s public relations campaign on “sick building syndrome” and ventilation as an alternative to smoke-free environments (Barnes et al. 1995; Drope et al. 2004). The proposed new study would generate personal sampling data for workplace and away-from-work SHS exposures of a large number of subjects for 24 hr in geographically diverse nonindustrial environments, providing the data the NIOSH Bulletin (NIOSH 1991) suggested in 1991.

### The origin of R.J. Reynolds’ role in the 16 Cities Study

Seven years earlier, in 1984, RJR Research and Development implemented a “Study Plan” (Colucci 1984) to provide RJR with data on exposure to SHS in response to growing public concern about SHS. The plan had two goals: *a*) to develop methods to quantitatively assess SHS exposures in homes, workplaces, and public places using chemical markers for SHS; and *b*) to develop “chemical indices of ETS components that could be measured in body fluids” (Colucci 1984). RJR’s new ETS research unit was led by Charles Green (Colucci 1985). Green was also a member of the TI’s ETS Advisory Group that reviewed and recommended funding of external research projects by the TI. Later, he served on the board of directors of the Center for Indoor Air Research (CIAR), the industry agency created to replace the ETS Advisory Group to fund external research on SHS (Barnes and Bero 1996).

In mid-1992, RJR Research and Development chemist Michael Ogden proposed to CIAR that it sponsor a nationwide SHS exposure assessment survey specifically for industry use in opposing any smoke-free workplace requirement in the anticipated OSHA indoor air quality standard (CIAR 1994; Green 1992, 1995a, 1997a, 1997b; Ogden 1997; OSHA 1995, 11614–11618).

CIAR described the proposed study to its board of directors as “a very significant enhancement over RJR nine city study which was recently presented to EPA SAB [Science Advisory Board] panel on ETS” (CIAR 1992; Davis and Stiles 1992), which was considering the then-proposed U.S. EPA risk assessment on SHS. The RJR nine city study compared reported smoking status by questionnaire with smoking status assessed by cotinine (Ogden et al. 1997); it did not have measurements of SHS exposure. The CIAR executive committee wanted to do the project, but its members identified two problems.

The committee believed there was no existing equipment to do the personal monitoring and no laboratory to conduct the chemical analyses (Green 1997b). Although RJR scientists had the technical expertise to do the laboratory analyses (OSHA 1995, 11659–11661; Wheeler 1988), the RJR laboratory capacity to handle such a large volume of work was not known. CIAR contacted scientists at Oak Ridge National Laboratory (ORNL) who had previously worked with CIAR on SHS research for a cost estimate to overcome these obstacles (Green 1997b; OSHA 1995, 11618–11619). Ogden offered that he and his colleagues at RJR would design and construct the personal sampling equipment, collect the samples, and make the analytical measurements for the proposed study (Green 1997b) with others collecting the data.

### The design of the 16 Cities Study

On 28 January 1993, RJR’s Ogden scheduled a meeting of RJR, Bellomy Research, Inc., ORNL, and CIAR to continue discussion of the study (Ogden 1993). Bellomy, a marketing research company, was a frequent contractor for human subject recruitment for RJR in-house scientific studies. The agenda shows that RJR was in charge of the “scope and objectives” of the study and that the four organizations would discuss and reach agreement on major components of the study, including the number of cities, number of participants, definition of cells, and restrictions on subject participation (Ogden 1993).

Roger Jenkins, an analytical chemist at ORNL, submitted the ORNL proposal requested by CIAR for the project on 16 February 1993 (Broin 1997, 14531–14532; Jenkins and Guerin 1993), and the CIAR board of directors approved $1.2 million in “directed study” funding for a 12 cities study project on 19 February 1993 (CIAR 1993, 1994; Heck 1993). A “directed,” “applied,” or “special-reviewed” project funded by CIAR was not peer reviewed by the CIAR scientific advisory board; these projects were used to meet the industry’s political and legal needs (Barnes and Bero 1996; Drope and Chapman 2001). CIAR issued separate contracts with RJR, Bellomy, and ORNL (Eisenberg 1993; Heck 1993; OSHA 1995, 9777–9778): for RJR, $360,000 for sampling equipment and materials, field sampling, laboratory analysis of samples, and compilation of raw data; for Bellomy, $480,000 for subject recruitment and selection; and for ORNL, $350,000 for quality assurance and control, and data interpretation and reporting. The overall manager of the project appears to have been CIAR executive director Max Eisenberg, whom Jenkins described to OSHA as his boss and the project officer (OSHA 1995, 9876–9877).

The study funded by CIAR involved recruiting 100 nonsmoking subjects in each of 12 cities distributed geographically across the United States (Ogden 1995). Subjects were to be recruited to populate equally each of 4 cells in a 2 × 2 design (Bailey 1993; Jenkins and Guerin 1993): smoking home/smoking workplace (SH/SW, Cell 1), smoking home/nonsmoking workplace (SH/NW, Cell 2), nonsmoking home/smoking workplace (NH/SW, Cell 3), and nonsmoking home/nonsmoking workplace (NH/NW, Cell 4).

To determine exposure to SHS, each subject wore a personal sampling pump during work and a separate personal sampling pump away from work for the balance of a 24-hr period. In addition, subjects kept a written diary of the number of cigarettes being smoked within 100 ft of them every hour during the air sampling. The samples were analyzed to determine SHS exposure based on three vapor phase (nicotine, 3-ethenylpyridine, myosmine) and five particulate phase SHS constituents (respiratory suspended particulates, ultraviolet absorbing particulate matter, fluorescing particulate matter, solanesol, scopoletin).

Salivary cotinine (a stable metabolite of nicotine) samples were obtained from each subject to confirm that they were nonsmokers and as a measure of the amount of nicotine inhaled (NRC 1986b; U.S. DHHS 1986; U.S. EPA 1992).

### The conduct of the 16 Cities Study

Bellomy designed and executed the plan for recruiting and selecting the human subject participants and selected the 12 cities with concurrence by RJR (Bailey 1993; Broin 1997, 14537–14540; OSHA 1995, 11666–11670). The selection criteria for cities included dispersion among the nine U.S. Census Bureau Regions and a variety of weather conditions (OSHA 1995, 11666–11670).

Field sampling for the original 12 cities study began on 13 May 1993 (Maiolo 1993), and was managed by Katherine Maiolo, an RJR chemist. Maiolo reported her field work directly to RJR’s Green. RJR conducted all of the laboratory analysis of the air and salivary cotinine samples (Broin 1997, 14531–14533; Green 1995a), with the air sample analyses under the direction of RJR’s Ogden, and the salivary cotinine samples were processed in a RJR biological chemistry research unit (OSHA 1995, 11604).

In a 28 December 1993 letter to CIAR’s Eisenberg (Jenkins 1993), Jenkins criticized some of Bellomy’s participant recruitment (failure to recruit equally for the four exposure cells) and complained about RJR’s “analytical difficulties” with salivary cotinine data from one city. Jenkins concluded that, as a result, the data set was limited in its usefulness, which would “restrict the generalization of the conclusions to a relatively narrow focus” (Jenkins 1993). After completion of the field sampling and analyses of the samples from the original 12 cities, the data set was not equally populated (25% per cell), but heavy on the non-smoking home/nonsmoking workplace data (Cell 4) with 686 (57%) subjects, and light on the smoking home/smoking workplace (Cell 1) with only 136 subjects (11%). Jenkins reported these problems to CIAR because “one of the most important objectives of this study is the relationship between time-averaged ETS exposure and salivary cotinine levels for individuals who live and work around smokers, compared with those who live in [*sic*] work in truly non-smoking environments, any loss of Cell 1 (SH/SW) participant data is significant” (Jenkins 1993). Jenkins suggested adding four more cities to the study, with particular emphasis on increasing the percentage of smoking home/smoking workplace (Cell 1) participants (Jenkins 1994e).

The CIAR board of directors approved an additional $440,000 to expand the study to 16 cities to obtain the additional data Jenkins had suggested (Heck 1994), but with the direction that a technical meeting be held between the CIAR board members and ORNL (Eisenberg 1994) “to finalize the experimental protocols to bring the ETS Exposure Study” to conclusion. Field sampling on the additional four cities was completed on 18 June 1994 (Maiolo 1994), bringing the total sample size to 1,564 people (507 males, 1,057 females).

### The OSHA proceedings

Jenkins and his ORNL supervisor Mike Guerin were identified as the co-principal investigators of the 16 Cities Study throughout the OSHA presentations (Jenkins 1994c; Jenkins and Guerin 1993, 1994, 1995a). A “Project Status and Summary” was presented to the OSHA staffers (Jenkins 1994d) on 16 March 1994 that identified all 16 cities, but included exposure data only for cities 1 through 6 and salivary cotinine data only for city 1. It showed exposures in the home 5–10 times higher than in the workplace for 6 of the 7 airborne exposure markers sampled and nearly twice for respirable suspended particles. The number of cigarettes that subjects reported being smoked in smoking work-places was reported; these showed increased levels of smoke constituents as the number of cigarettes reported being smoked increased. However, the smoking workplaces category included workplaces in which no smoking was observed by subjects.

Less than a month later, on 5 April 1994, OSHA issued its Notice of Proposed Rule-making on Indoor Air Quality (OSHA 1994) to adopt a workplace safety and health standard that included extensive restrictions on smoking in the workplace (Bryan-Jones and Bero 2003; OSHA 1994). The public was invited to submit formal comments on the proposed rule, with an 13 August 1994 deadline. These comments would form the basis for the planned public administrative hearing on the proposed rule.

Jenkins and Guerin submitted their Interim Report No. 3 (Jenkins and Guerin 1994) based on data from the first 12 cities to CIAR on 10 August 1994, just in time for CIAR to submit it to OSHA as CIAR’s comment. Interim Report No. 3 stated that it appeared the workplace was not the dominant source of SHS exposure, and that it is inaccurate to assume that workplace and residential exposures are comparable.

Jenkins also filed a comment (Jenkins 1994b) challenging OSHA’s measure of SHS exposure because OSHA considered only duration of exposure to SHS without considering SHS concentration, and noting that the pending 16 Cities Study would provide the first determination of personal exposure to SHS constituents.

When Jenkins presented the 16 Cities Study results at the OSHA hearing, he characterized it as the most representative study of U.S. workplaces that had ever been undertaken (Jenkins and Guerin 1995b). Later, RJR lead SHS scientist Green defended (Green 1997a) the 16 Cities Study (Jenkins et al. 1996) in a response to a journal article criticizing CIAR funding of tobacco-related research such as the 16 Cities Study (Barnes and Bero 1996); Green described the 16 Cities Study as the only authoritative SHS study OSHA had before it for consideration on the proposed Indoor Air Quality Standard and “the largest, most relevant, and most representative study of its kind, using the most appropriate analytical methodology” (Green 1997a). Green identified himself in the response as an RJR scientist and CIAR Board member, but he did not disclose his extensive role in origin and conduct of the study.

### Omitted data on the amount of smoking near study subjects

During Jenkins’ presentation at the 5 January 1995 OSHA hearing, Matt Myers, representing public health organizations at the hearing, asked Jenkins about the determinants of the level of SHS described in *The Chemistry of Environmental Tobacco Smoke: Composition and Measurement* (Guerin et al. 1992), a book on SHS that CIAR commissioned Jenkins and Guerin to write. Jenkins agreed that one of the relevant determinants of SHS exposure he and Guerin described was the number of cigarettes being smoked in the area being studied (OSHA 1995, 9904–9911). None of the data submitted to OSHA from the completed 16 Cities Study (Jenkins 1994a, 1994c, 1995a; Jenkins and Guerin 1994), however, included the data (Bellomy Research 1993a, 1993b) on the number of cigarettes being smoked within 100 ft of the experimental subjects each hour, which show no smoking or relatively little smoking occurring in many workplaces designated as smoking workplaces in the study. Myers asked why these data were omitted. Jenkins responded that the raw data in the diaries were self-reported observations and expressed skepticism about their value (OSHA 1995, 9904–9911).

In 1997, however, a study (Sapphire Group 1997) using the 16 Cities data set for CIAR by the Sapphire Group, a risk assessment consulting firm (Tardiff 1996, 1997, 1998), reported that the 16 Cities data showed a significant association between workplace concentrations of SHS components and the number of cigarettes being smoked.

In 1999, Jenkins coauthored a paper (LaKind et al. 1999) with two people from the Sapphire Group and others using the 16 Cities data on the number of cigarettes being smoked, and concluded that the number of cigarettes smoked had an impact on the SHS exposures measured. Jenkins did not comment on why the data on the number of cigarettes being smoked at work—which he had viewed skeptically at the OSHA hearing (OSHA 1995, 9904–9911) and omitted from his original publication of the 16 Cities Study (Jenkins et al. 1996)—were now worthy of publication.

### The posthearing period

After Jenkins’ 5 January 1995 presentation at the OSHA Hearing, Green (1995b), the RJR representative on the CIAR Board and manager of RJR’s component of the 16 Cities Study, wrote his fellow board members on 13 April 1995, recommending that the CIAR board assemble a team of knowledgeable scientists to review with Jenkins his 5 January 1995 OSHA presentation. Jenkins summarized the recommendations made by the CIAR scientific team to change how the data were to be presented in the future (Jenkins 1995b). All of the changes recommended by the CIAR scientific team appeared in Jenkins’ posthearing comments (Jenkins 1995a).

## Discussion

Secondhand tobacco smoke is the major source of indoor air pollution in the United States and globally, surpassing most other sources for particulate matter, volatile organic compounds, and most toxic chemicals in the home and the nonindustrial workplace, except in some developing countries where biomass fuels are burned indoors for heating and cooking. The 16 Cities Study is one of the largest studies ever conducted of occupational exposures to SHS. Understanding the limitations of the study, and what information can be gleaned from the study, are of great value to all concerned about indoor air pollution.

The 16 Cities Study reported that exposures in homes with unrestricted smoking were two to four times higher than exposures in workplaces where smoking was allowed, and concluded that exposures in the workplace were only 30–60% of those estimated by OSHA from residential exposure data for average workers, and only 15–20% for the most highly exposed workers. OSHA had based its risk assessment on residential SHS exposure risk because of the acknowledged lack of research on the concentration and distribution of SHS components in the work-place (NIOSH 1991; OSHA 1994) by assuming that residential and workplace exposures to SHS were comparable. The 16 Cities Study challenged this assumption because it provided workplace SHS exposure data and compared that exposure to at-home exposures for the same person. In addition, the 16 Cities Study is particularly important because its widespread use in legislative and regulatory hearings to substantiate the tobacco industry’s long-standing argument that smoking is not a major source of indoor air pollution (Bryan-Jones and Bero 2003; Drope et al. 2004).

Assuming that the 16 Cities data are valid, however, a more appropriately formulated analysis results in the opposite conclusions.

### Definition of a “smoking workplace.”

Although in his testimony to OSHA Jenkins classified workplaces dichotomously—either smoking or not smoking—smoking was not permitted everywhere in most “smoking work-places.” Included, but not emphasized, in the written submissions Jenkins presented to OSHA (Jenkins 1994a; Jenkins and Guerin 1994, 1995a) were data that indicated that > 68% of the 16 Cities Study’s “smoking workplaces” restricted smoking to designated smoking areas only. The same data (Jenkins 1994a, Table 6) show that median concentrations of nicotine in the restricted smoking workplaces was 0.088 μg/m^3^ compared with 0.575 μg/m^3^—6.5 times greater—in “smoking workplaces” with no restrictions on smoking, although he did not highlight this point. Hammond et al. (1995) reported a median nicotine concentration in offices that restricted smoking of 1.3 μg/m^3^, compared with 8.6 μg/m^3^ in offices without restrictions—a ratio of 6.6, remarkably similar to the 6.5 found in the 16 Cities data. The 1996 16 Cities Study paper (Jenkins et al. 1996) made no distinctions between workplaces that did and did not restrict smoking to designated areas, even though smoking restrictions reduce SHS concentrations significantly. Jenkins and Counts (1999) did present a more detailed breakout of the unrestricted and restricted smoking workplace data in 1999, although they neither highlighted nor discussed the different concentrations observed.

**Figure 1:**
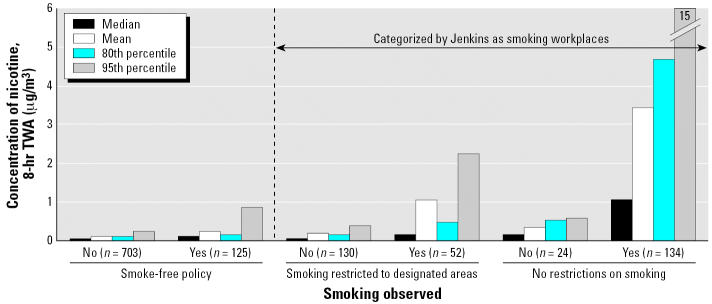
Effect of smoking policy and practice on the SHS exposures of workers. TWA, time-weighted average. The concentrations of nicotine observed among personal samples collected while at work varied with smoking policy. Smoke-free policies led to much lower concentrations of SHS than policies that restricted smoking to designated areas, but such restrictive policies did reduce SHS concentrations from the levels observed in workplaces without any policies restricting smoking. The categorization by Jenkins et al. (1996) of workplaces that restricted smoking to designated areas as “smoking workplaces” diluted this pool and so substantially reduced the reported SHS concentrations in “smoking workplaces.” Because over half the “smoking workplaces” in fact restricted smoking and the SHS concentrations where smoking was not restricted were > 6 times greater than where they were restricted, the mean reported was half the value that would have been observed had the correct categorization been used (including workplaces that allowed smoking without restrictions). Data from Jenkins and Counts (1999, Table 2).

The 16 Cities data, as we present them in Figure 1, make two observations readily apparent: First, fewer than half (47%) the data from “smoking workplaces” are drawn from workplaces that have no restrictions on smoking. Second, SHS concentrations are much higher where there are no restrictions than in workplaces that restrict smoking (3.4 compared with 1.1 μg/m^3^ mean nicotine, when smoking is observed). Thus, even though the study was designed to have 50% of its sample people who worked in smoking workplaces, only 10% worked where smoking was allowed without restriction. Another 12% worked where smoking was restricted to designated areas, and less than a third of these subjects observed smoking at any time during the day. Moreover, those who did observe smoking reported fewer cigarettes than reported by those from workplaces without restrictions. These observations hold if other tobacco-specific markers (e.g., 3-ethenylpyridine and myosmine) are examined. Jenkins himself reported this problem of too few subjects with ETS exposure at work to CIAR’s Eisenberg in 1993 (Jenkins 1993), early in the conduct of the study; he did not highlight it in the public reports of the results, even though this serious problem persisted.

### Inappropriate comparisons among the cells

One of the key conclusions in the Jenkins et al. (1996) 16 Cities paper is, “For the majority of subjects who either lived or worked in smoking environments, the home was found to be the greater source of ETS exposure.” This conclusion is based on a comparison of exposures of those who live with smokers to those who work with smokers. However, the more relevant question to the OSHA proceedings (and any consideration of smoke-free workplace policies) is whether workplace exposure to SHS adds significantly to the total SHS exposure experienced by individuals. Rather than grouping all the data, Jenkins et al. (1996) could have stratified the data by the home smoking status and then reported the relative exposures of those who work in smoking environments and those who work in non-smoking environments.

**Figure 2:**
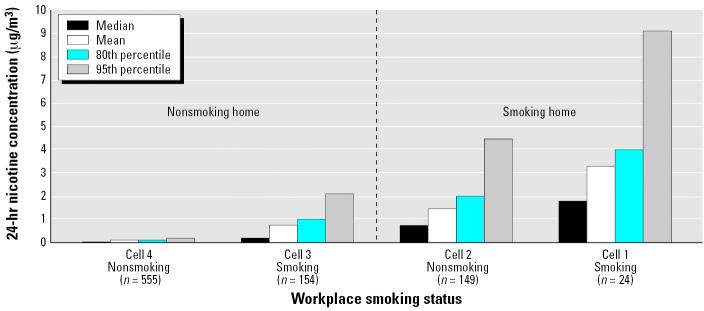
Daily average concentrations as a function of smoking policies at home and at work. (Smoking workplaces include those that restrict smoking to designated areas.) The 24-hr concentrations are the average of the 8-hr “at work” and the 16-hr “away from work” personal samples. The daily average concentrations increase dramatically for those who live in nonsmoking homes if they work in an environment that “allows smoking” (compare Cell 2 to Cell 1). Those who live in smoking homes also experience a large increase in daily exposures if they also work where smoking is allowed or restricted (compare Cell 4 to Cell 3). Clearly whether or not one works where smoking is allowed has a significant impact on the total daily exposure. Other tobacco specific markers of SHS demonstrate similar relationships. Data from Jenkins et al. (1996, Table 6).

Although not making this comparison themselves, Jenkins et al. (1996, Table 6) do provide data appropriate for this analysis, which we plot in Figure 2. Among those who live in smoking homes, working in a confirmed smoking workplace doubled their total 24-hr average SHS exposures (median, mean, 80th, and 95th percentiles) compared with those who worked in nonsmoking environments. The effect is even more pronounced for those who live in nonsmoking homes: Their total exposures are > 10 times higher if they work in smoking environments compared with those working in nonsmoking environments. On an absolute scale, those exposed only at work (Cell 3, NH/SW) experience, on average, half the 24-hr exposure as those exposed only at home (Cell 2, SH/NW), but this result reflects primarily the fact that twice as much time is spent away from work as at work (16 vs. 8 hr/day). These data indicate that if workplaces were smoke-free, the total SHS exposure of those living with smokers could be cut in half, and the total SHS exposure of those living in nonsmoking homes would become negligible, a significant worker safety and public health benefit.

### Real roles of the parties in the 16 Cities Study

Table 2 compares the roles of CIAR and its contractors in conducting the 16 Cities Study described by Jenkins in the 16 Cities Study (Jenkins et al. 1996). The most significant deviation between Jenkins’ description and the actual situation is that Jenkins’ description seems to indicate that ORNL had overall control of the study design and implementation, even though these critical functions were controlled by RJR and CIAR (Table 2). The published paper (Jenkins et al. 1996) did acknowledge the RJR scientists “for their outstanding contributions to laboratory and field operations,” but they were not listed as co-authors. In contrast, RJR’s Green wrote in 1997 in an internal RJR memo supporting a promotion for Ogden: “The so-called ‘16-Cities Study’ was published by ORNL scientists in a peer-reviewed journal. Because of the political beliefs of some sponsors, Dr. Ogden and his colleagues were acknowledged in the paper for their contributions, but were not granted their rightful recognition as coauthors” (Green 1997b; Ogden 1997).

In 1997 Jenkins did fully describe the extensive role that RJR played in designing the 16 Cities Study in the secondhand smoke lawsuit (Broin 1991) brought against tobacco companies by airline flight attendants exposed to in-flight SHS before smoking was eliminated on all U.S. flights (Broin 1997, 14518–14564; Wilson 1997). Tobacco industry lawyers wanted to use Jenkins and the 16 Cities Study to show that the flight attendants were exposed to SHS levels that were too low to account for any of the health problems the flight attendants were claiming resulted from in-flight exposure.

After a hearing in chambers in which Jenkins described the extensive role that RJR played in designing the 16 Cities Study, obtaining the field sampling data and analyzing them, and the limited role Jenkins and his ORNL colleagues played in data collection, Judge Robert Paul Kaye concluded that Jenkins would not be allowed to testify concerning his published 16 Cities Study because RJR’s extensive involvement in the study raised concerns about its reliability (Broin 1997, 14570–14571; Daubert 1993; General Electric 1997).

Jenkins and colleagues later published several papers using the 16 Cities data set that showed that the number of cigarettes observed in the workplace had an impact on SHS exposure concentrations (LaKind et al. 1999), that restricting smoking to designated areas reduces nonsmoker SHS exposures (Jenkins and Counts 1999), and that making workplaces nonsmoking eliminates the great majority of nonsmoker SHS exposures (Graves et al. 2000). It is important to emphasize, however, that this work was published long after the close of the OSHA proceedings, and influencing OSHA was the primary reason for the 16 Cities Study.

### The broader implications of the 16 Cities Study

Although the results of industry-funded research consistently reflect a bias in support of the sponsors’ interests (Bekelman et al. 2003; Bero 2005a; Bero and Rennie 1996; Bhandari et al. 2004; Boyd and Bero 2000; Boyd et al. 2003; Cho and Bero 1996; Glaser and Bero 2005; Levine et al. 2003; Lexchin et al. 2003; Lipton et al. 2004; Martinson et al. 2005; Yaphe et al. 2001), it is only because of the public availability of millions of pages of internal tobacco industry documents that we can examine the mechanics of the tobacco industry’s efforts (Bero et al. 2005; Bitton et al. 2005; Malone and Bero 2003; McKee 2003; Shamasunder and Bero 2002; Tong et al. 2005) to develop and present scientific results specifically designed to support its corporate advocacy efforts. This detailed understanding leads to some principles that can be applied generally. Research funded by an industry seeking to affect regulation should be reviewed and analyzed very critically, particularly for subtle forms of influence on the presentation of results. Journals should require not only disclosure of funding but also disclosure of the involvement of the sponsor in the conduct of the research and preparation and revision of the resulting papers, such as already required by *The Lancet* (Lancet 2006).

## Conclusion

The 16 Cities Study, as with the conclusions of the 1998 aircraft cabin air quality study conducted for the tobacco industry (Neilsen and Glantz 2003), offers an example in which researchers funded by the tobacco industry minimized the involvement of the industry in the actual conduct of a research project and presented the results in a way that supported the industry’s political or legal position. The 16 Cities Study was specifically conceived and designed to forestall regulation of workplace smoking. The extensive involvement of RJR, in particular, in the design and execution of the study, was never clearly disclosed in any of the publications or public presentations of the results. The study authors combined exposure data from restricted and unrestricted work-places and compared exposure data among study cells in an inappropriate manner. That analysis produced results the industry could cite to support its claim that workplace SHS exposures were low compared with household exposures during its efforts to defeat indoor smoking restrictions. In fact, an alternative presentation of the same data (Figure 2) demonstrates significant workplace secondhand smoke exposures and supports the need for smoke-free workplaces.

## Figures and Tables

**Table 1:** 16 Cities Study timeline.

Date	Government or public health action	Tobacco industry action
1981	Hirayama study on lung cancer in nonsmoking wives of Japanese smokers published (Hirayama 1981)	
1984		RJR R&D implements SHS Study Plan (Colucci 1984)
1986	Surgeon General issues report on involuntary smoking (U.S. DHHS 1986) NRC issues report on measuring exposures and assessing health effects of SHS (NRC 1986b) NRC issues study on air quality and safety in airliner cabins (NRC 1986a)	
1987	Congress passes law ending smoking on short airline flights Citizen petition filed seeking emergency OSHA standard on workplace smoking (Public Citizen 1987)	
1988		Philip Morris promotes tobacco industry study of in-flight air quality
	Consultant recommends to OSHA it conduct personal sampling study of workplace SHS exposure (Meridian Research 1988)	
1989	OSHA denies citizen petition	
		Tobacco industry’s in-flight air quality study published (Malmfors et al. 1989)
1991		Tobacco Institute Executive Committee approves U.S. EPA/OSHA Strategic Plan
	NIOSH issues Current Intelligence Bulletin on SHS (NIOSH 1991)	
		Tobacco Institute Executive Committee adopts U.S. EPA and OSHA scientific research plan
		Tobacco industry funds Japanese spousal study to challenge Hirayama findings
	OSHA publishes Notice of Request for Information on Occupational Exposure to Indoor Air Pollutants (OSHA 1991)	
1992		RJR proposes nationwide personal sampling SHS exposure assessment to Center for Indoor Air Research (CIAR) CIAR recruits Jenkins and Guerin of Oak Ridge National Laboratory to front for nationwide SHS study
	EPA publishes its risk assessment *Respiratory Health Effects of Passive Smoking: Lung Cancer and Other Disorders* (U.S. EPA 1992)	
1993		CIAR funds 12 Cities Study for nationwide SHS exposure assessment RJR scientists conduct fieldwork and laboratory analysis for 12 Cities Study Jenkins complains to CIAR about shortcomings in data and recommends adding 4 more cities to study
1994		CIAR funds additional 4 cities for study Jenkins and Guerin present preliminary findings for cities 1–6 to OSHA staff
	OSHA issues its Notice of Proposed Rulemaking on Indoor Air Quality	
1995		RJR scientists conduct field work and laboratory analysis for 4 additional cities Jenkins submits Comments on Proposed Rulemaking to OSHA with data from first 12 cities (Jenkins 1994b) CIAR submits ORNL Interim Report No. 3 covering cities 1–12 to OSHA (Jenkins and Guerin 1994) Jenkins submits Addendum to Comments on Proposed Rulemaking updating data to include all 16 cities (Jenkins 1994a) Jenkins testifies at OSHA Hearing on Indoor Air Quality Standard Japanese spousal study journal article published (Lee 1995)
1996		16 Cities Study journal article published (Jenkins et al. 1996)
1997	16 Cities Study rejected as unreliable by Judge Kaye in flight attendants’ SHS litigation (Broin 1997)	
1999		Journal article published on 16 Cities smoke density data (number of cigarettes being smoked) omitted from original 16 Cities Study (LaKind et al. 1999)
2001	OSHA withdraws Notice of Proposed Rulemaking on Indoor Air Quality	

Abbreviations: CIAR, Center for Indoor Air Research; ETS, environmental tobacco smoke; ORNL, Oak Ridge National Laboratory; R&D, research and development; TI, Tobacco Institute.

**Table 2:** Functions of organizations in the conduct of the 16 Cities Study.

Function	ORNL	RJR	BRI	CIAR
Overall conduct of the study	*			A
Development of study design	P	A		
Selection of outside firms	P	A^a^	A^b^	A^c^
Oversight of field sampling	P, A			
Quality assurance	P, A			
Data study and interpretation	P, A	A		A
Reporting of results	P, A			A^d^
Contracting with outside firms^e^				A
Recruitment of human subjects			P, A	
Conduct field operations		P, A	P, A	
Conduct information coding			P, A	
Provide human subject data to ORNL			P, A	
Provide sampling material and equipment		P, A		
Analyze field samples		P, A		
Compile analytical data		P, A		

Abbreviations: A, as actually performed; BRI, Bellomy Research, Inc; P, as reported by Jenkins et al. (1996) in original published paper; R&D, research and development;

* function is listed in ORNL’s original proposal to CIAR (Jenkins and Guerin 1993) as an ORNL function, but the function is omitted entirely from the paper.

a Selected Bellomy Research, Inc. to recruit human subjects.

b Selected local marketing research firms for human subjects recruitment.

c Selected RJR R&D to conduct field sampling, analyses of field samples, and compilation of analytical data.

d CIAR directed several changes in reporting of results in the final paper (Jenkins et al. 1996).

e Function is listed in ORNL’s original proposal to CIAR as a CIAR function (Jenkins and Guerin 1993), but omitted entirely from the paper (Jenkins et al. 1996).
